# Sequencing the mosaic genome of Brahman cattle identifies historic and recent introgression including polled

**DOI:** 10.1038/s41598-018-35698-5

**Published:** 2018-12-10

**Authors:** L. Koufariotis, B. J. Hayes, M. Kelly, B. M. Burns, R. Lyons, P. Stothard, A. J. Chamberlain, S. Moore

**Affiliations:** 1 Centre of Animal Science, Queensland Alliance for Agriculture and Food Innovation, The University of Queensland, Brisbane, Queensland 4072 Australia; 2 Australian Agricultural Company (AACo), Brisbane, Queensland 4006 Australia; 3 Department of Agriculture and Fisheries, Rockhampton, Queensland 4702 Australia; 4 School of Veterinary Science, The University of Queensland, Gatton, Queensland 4343 Australia; 5 Department of Agricultural, Food and Nutritional Science, University of Alberta, Edmonton, Alberta T6G 2C8 Canada; 6 Agriculture Victoria, Agribio, Centre for Agribiosciences, 5 Ring Road, Bundoora, Victoria 3086 Australia

## Abstract

Brahman cattle have a *Bos indicus* and *Bos taurus* mosaic genome, as a result of the process used to create the breed (repeat backcrossing of *Bos taurus* females to *Bos indicus* bulls). With the aim of identifying *Bos taurus* segments in the Brahman genome at sequence level resolution, we sequenced the genomes of 46 influential Brahman bulls. Using 36 million variants identified in the sequences, we searched for regions close to fixation for *Bos indicus* or *Bos taurus* segments that were longer than expected by chance (from simulation of the breed formation history of Brahman cattle). Regions close to fixation for *Bos indicus* content were enriched for protein synthesis genes, while regions of higher *Bos taurus* content included genes of the G-protein coupled receptor family (including genes implicated in puberty, such as *THRS*). The region with the most extreme *Bos taurus* enrichment was on chromosome 14 surrounding *PLAG1*. The introgressed *Bos taurus* allele at *PLAG1* increases stature and the high frequency of the allele likely reflects strong selection for the trait. Finally, we provide evidence that the polled mutation in Brahmans, a desirable trait under very strong recent selection, is of Celtic origin and is introgressed from *Bos taurus*.

## Introduction

Brahman cattle are an interesting model for studying the impact of natural and artificial selection on the genome. Between 1854 and 1926 four *Bos indicus* cattle breeds (Ongole, Krishna, Gir and Gujarat) from India, that were highly adapted to tropical conditions, were imported into the USA. The Brahman breed was created by repeated crossbreeding of these animals with local *Bos taurus* cattle to generate large numbers and “grade up” to *Bos indicus*^1^. In 1933, Brahman bulls were first imported into Northern Australia, and the Brahman cattle were again “graded up” using local *Bos taurus* breeds. Brahman cattle therefore have a mosaic *Bos indicus* and *Bos taurus* genome, with around 10% of the genome of modern Australian Brahman cattle of *Bos taurus* origin^2^. Brahman cattle today are grazed for beef production in harsh conditions in Northern Australia, so are under strong selection for adaptation to these environments. *Bos indicus* and *Bos taurus* regions of the genome can be readily identified, so we can ask the question are there segments of the genome that are Bos taurus in origin and longer than expected by chance (given the history of the breed). This would be evidence for selection in these regions and could lead to identification of causative loci.

Whole genome sequencing should be a powerful method to identify such regions if they exist, provided enough individuals of the mosaic population are sequenced and sequence data is available on the contributing populations. This approach was recently used to provide evidence that Mongolian yak (*Bos grunnies*) has introgressions from *Bos taurus*, finding that Mongolian yaks have inherited 1.3% (on average) of their genome from bovine and the introgressed regions are enriched for genes associated with nervous system development and function (related to aggression)^3^. The authors also demonstrated that a polled mutation segregating in Mongolian yaks was *Bos taurus* in origin.

Our aim here was to identify *Bos taurus* segments in the Brahman genome at sequence level with whole genome sequence data and then to hypothesize if there are regions of the genome that have been under artificial or natural selection, and further some of these can be identified because they result in long segments that are *Bos taurus* in origin. While *Bos indicus* cattle are well adapted to harsh environments, they can have reduced production performance compared to *Bos taurus* cattle, including slower growth and reduced fertility, particularly later puberty and stronger expression of post-partum anoestrus^4^. This suggests an additional hypothesis that we test; that the Brahman genomes could be enriched for *Bos taurus* segments in regions affecting these traits, reflecting some selection during the grading up process.

To look for regions of complete or nearly complete *Bos indicus* or *Bos taurus* fixation in the Brahman population, we sequenced the genomes of 46 Brahman bulls used in Australia between 1953 and the present day. These bulls were selected because they were key ancestors of the modern Brahman population. *Bos indicus* and *Bos taurus* content was assessed in small fixed-size windows using a *bosind****_****250* calculation across the genome using allele frequencies of the sequence derived SNP.

In this study, we found that alleles at genes in the G-coupled receptor family, immunity and a range of beef productivity traits are likely of *Bos taurus* origin^5^. As a demonstration of the power of the method, we also investigated the origin of polledness (lack of horns) in Brahmans, a trait which has recently become very valuable for welfare and safety reasons^6^. Using whole genome sequence from three homozygous polled Brahman cattle we found evidence that at least in some Brahman cattle the polled mutation is of *Bos taurus* origin, specifically the Celtic mutation^7,8^.

## Results and Discussion

**Figure 1:**
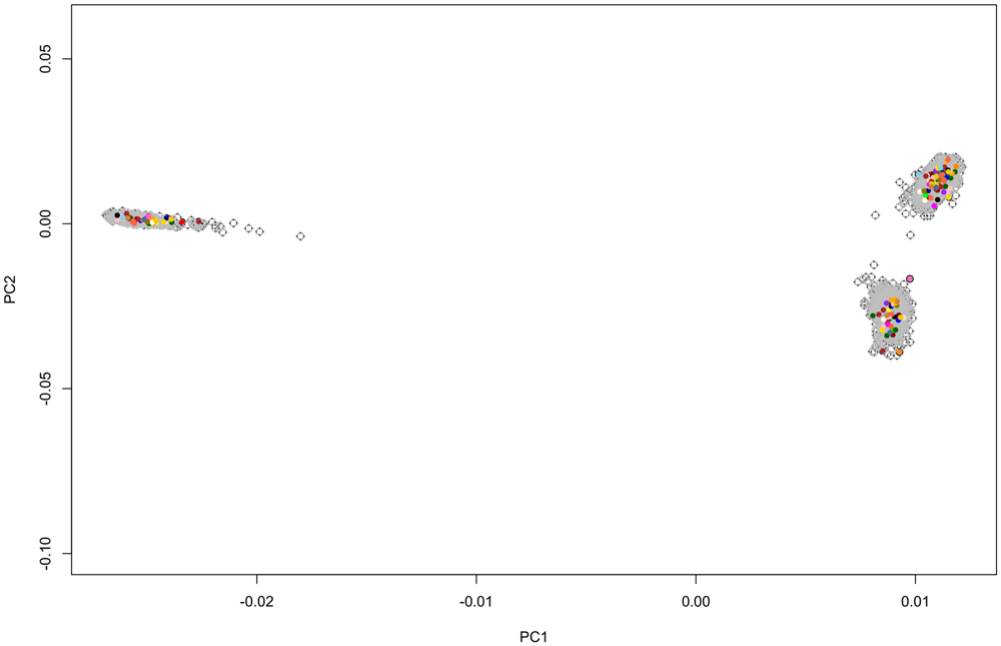
Principle component analysis of the genomic relationship between studs. Forty-six bulls selected for sequencing (in colour) relative to the diversity of the Brahman population represented by a large sample of genotyped animals with 24 K SNP (1021 animals in grey). PC1 and PC2 are the first and second principle components of genomic relationship matrix among the genotyped animals.

Bulls were selected for sequencing using an algorithm to identify 46 bulls that captured the highest proportion of genetic variation in the breed, based on an analysis of an extensive Brahman pedigree and a stepwise regression procedure^9^. This selection method avoided double counting of ancestral genomes and considered whether DNA, extracted from semen straws or Ampules, was available for a bull or not^10^ (Fig. 1). The 46 bulls selected captured 17% of the genetic variation represented in the pedigree. The selected bulls were sequenced on an Illumina HiSeq sequencer^11^, at an average of 12.5 times genome coverage, with a range of 10 to 30 times genome coverage.

The genome sequences were aligned to the UMD3.1 reference genome and variants were called using GATK^12^. After filtering (see Methods), a total of 36,114,110 SNP and 4,779,538 Indels remained. This represents a much higher rate of polymorphism than is observed in *Bos taurus* breeds^10^ for the same number of animals sequenced. The higher rate of polymorphism in Brahmans is likely a reflection of a larger ancestral population size for *Bos indicus* than *Bos taurus* cattle (pre-domestication)^13^, the fact that four *Bos indicus* breeds were used to generate the breed, as well as the infusion of *Bos taurus* into Brahmans during breed formation.

Annotation of the variants with the NGS-SNP pipeline^14^ indicated that the majority the variants detected were intergenic. Of the intragenic variants 0.35% were missense mutations and only a small number of stop gained mutations were annotated (see Supplementary Table S1).

### Shared variants between the cattle sub-species

Of the Brahman foundation indicine breeds, whole genome sequence was available for Gir that came from fourteen Gir animals (11 were used to build pooled samples and 3 individuals animals were sequenced individually)^15^. The Gir sequences available were taken as a representative of pure *Bos indicus*. However, we must acknowledge that this is a limitation in this analysis as we do not have other indicine breeds to build a more complete *Bos indicus* reference set of SNP. For *Bos taurus*, we used SNP allele frequencies from the 1000 bull genomes project, with 234 bull sequences of *Bos taurus* origin, that includes 129 Holstein-Friesians, 43 Fleckvieh and 15 Jersey breed cattle^10^.

**Figure 2:**
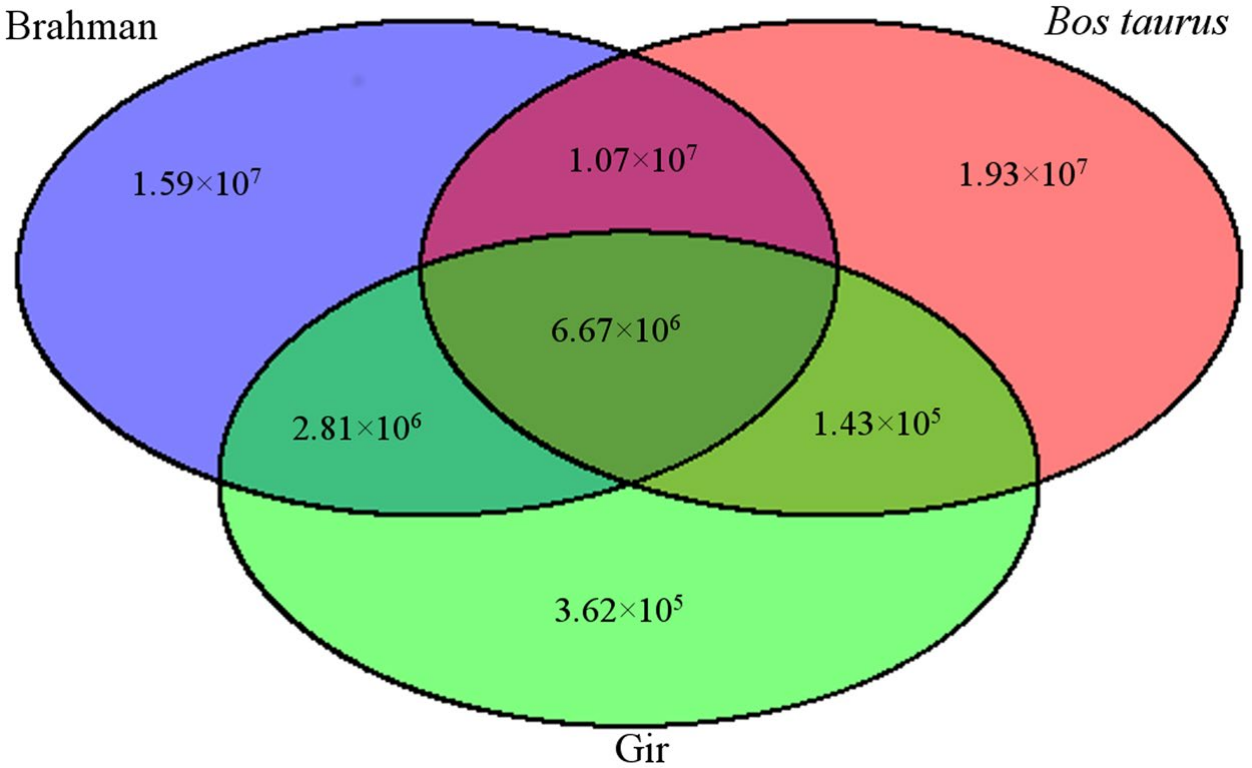
Common variants between the cattle breeds. The blue circle represents the Brahman variants, red represents Bos taurus and green represents all Gir variants. We find that overall, most of the Gir variants are shared with Brahman breeds and only a small proportion of variants are common between all three groups.

The number of Brahman variants that were in common with variants from *Bos taurus* and Gir were examined. Of the 36.1 million Brahman variants, 15.9 million variants were uniquely found in Brahman when compared with the other 2 datasets (Fig. 2). Most of these variants are likely to be from the other three indicine foundation breeds (Ongole, Gujarat and Krishna) and due to the limitation of using only one sequenced reference indicine breed. 10.7 million variants were shared exclusively between Brahman and *Bos taurus* (and not with Gir), meaning 48% of *Bos taurus* variants were found only in the Brahman genome, and not the Gir genome. There were only nine million SNP in the Gir dataset, reflecting the small number of Gir animals sequenced, however, 95% of variants from Gir were in common with the Brahman variants (expected as Gir was a founding breed). Overall a total of 6.7 million variants (18.5% of Brahman variants) were in common between all three datasets.

### Brahman bull genome regions high in indicine content

To identify regions of the genome in each animal that were indicine or taurus in origin, we calculated *bosind_250* values (Methods) using a formula that is based on the ***b*** values from Bolormaa *et al*.^2^ *bosind_250* statistic is an estimate of comparing two populations based on their sequence, allele frequencies and SNP calls to determine how differentiated the two populations are. *bosind_250* values for each sequenced Brahman bull with both the Gir genomes and the *Bos taurus* genomes were calculated for SNP in 250 kb fixed-size windows (adjacent and non-overlapping). We used 250 kb windows as this meant enough SNP were in each window to allow for a robust estimate of the *bosind_250* value. A low *bosind_250* value in a 250 kb fixed-size window between Brahman and *Bos taurus* indicates that segment is of *Bos taurus* origin. A low *bosind_250* value between Brahman and Gir indicates that the segment is of Gir (*Bos indicus)* origin.

The global *bosind_250* value (across all SNP) for the Brahmans was substantially lower for *Bos indicus* (Gir, 0.188) than for *Bos taurus* (0.264), reflecting the high indicine content of Brahman genomes. There was substantial variation between the Brahman animals regarding the proportion of the genome that was indicine derived, indicating both global *bosind_250* and the proportion of fixed-size windows that had a lower *bosind_250* values with *Bos indicus* than *Bos taurus*. For example, sire 13 had an average *bosind_250* value of 0.26 with *Bos taurus*, and 0.24 for *Bos indicus*, with the fixed-size window analysis related to almost 50/50 *indicus*/*taurus* composition. In contrast, sire 1 had a *bosind_250* value of 0.27 for *Bos taurus* and 0.17 for *Bos indicus*, showing much higher *indicus* content. Averaged over all Brahman animals, 8.94% of the Brahman genome was taurine derived, which is slightly lower than the 10% previous estimate^2^, perhaps because we included several sires imported directly from the USA. Supplementary Fig. S1 shows each sequenced Brahman animal with its taurine/indicine percentage.

The difference in the percent of *bosind_250* values for each 250 kb window between Brahman/*Bos indicus* and Brahman/*Bos taurus* was calculated and a heatmap was generated for all chromosomes. These heatmaps are presented in a data repository hosted by LabArchives LLC, (see Data availability). Overall, the heatmaps show that much of the Brahman genome is largely of *Bos indicus* origin with only a few segments showing strong *Bos taurus* introgression. Chromosomes 8, 12, 14, 23, 26 and 29 all show regions in the genome that are of strong *Bos taurus* introgression. This will be discussed below.

**Figure 3:**
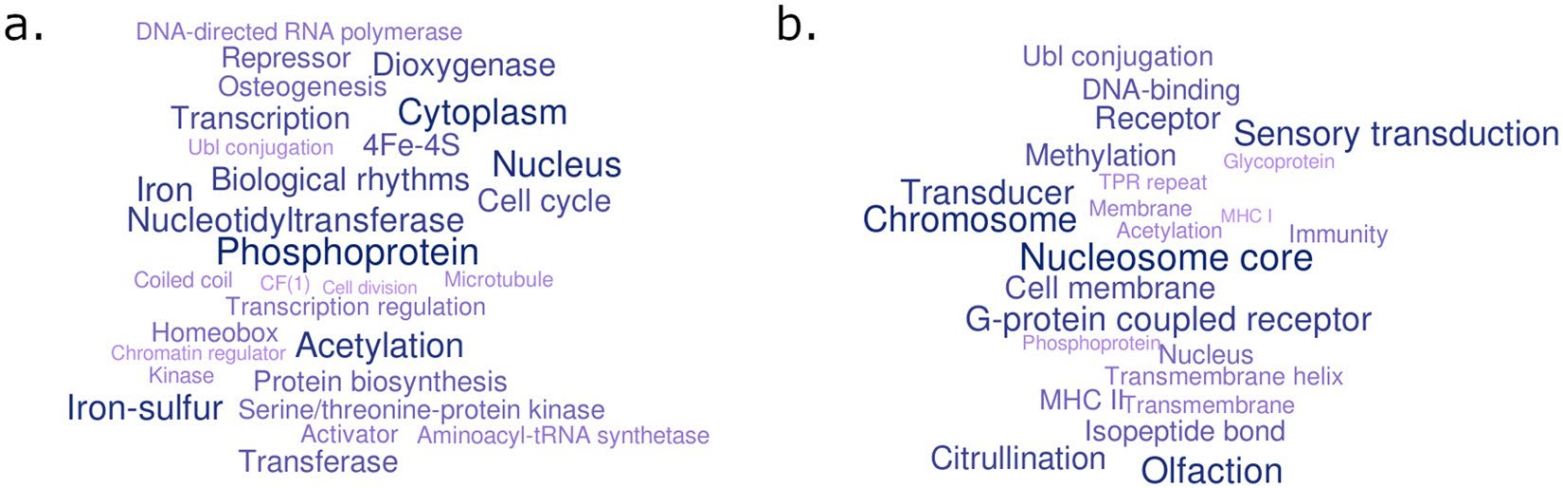
Word cloud showing the keywords after functional categorising genes using DAVID. Larger keywords represent those that show the strongest associations for that set of genes (based on the DAVID p-value). (**a**) Functional categorised genes that are in genomic segments that are homozygous or nearly homozygous in the sequenced bulls and of Bos indicus origin (**b**). Functional categorised genes located in genomic segments that show strong Bos taurus introgression.

Fixed-size windows were sorted in ascending order based on the percent difference in the *bosind_250* values between *Bos indicus* and Brahman and selected the top 5% windows that had the greatest percent difference (this was 368 fixed-size windows). To determine if these windows were enriched for gene pathways or annotations, all genes found in these fixed-size windows were extracted. This resulted in 1,609 unique genes in the regions of nearly fixed *Bos indicus* origin. Supplementary Table S2 provides the full list of the top 5% most significant fixed-size windows including all genes and gene information found within each fixed-size window. These genes were investigated for enrichment of function using the DAVID package^16,17^. Results from that analysis reveals there is significant enrichment for genes involved in intermediate filament, hormone, protein biosynthesis, cytoplasm and phosphoproteins (Fig. 3a). Protein biosynthesis (Fig. 3a) is interesting as there is speculation that Brahmans are more able to cope with harsh conditions because of lower protein turnover. We found 61 genes to be enriched for protein biosynthesis, including, but not limited to, G elongation factor mitochondrial 1 (*GFM1*), GUF1 homolog, GTPase (*GUF1*) and many eukaryotic translation elongation factors. Eukaryotic translation elongation factors and eukaryotic translation initiation factors are key regulators of protein synthesis^18^, protein bond formation^19^, delivery of tRNAs to translating ribosomes^20^, and proliferation (mainly *eEF2*) during skeletal muscle contraction^21^. To determine if the level of enrichment for the above functions could occur by chance, we performed a permutation test by randomly selecting the same number of genes (1,609 genes) as those found in the top 5% most significant *Bos indicus* regions. We performed this random selection process a total of 100 times and functional annotation of each permutation was performed with DAVID. The keywords for each permutation were recorded and grouped together to create a word cloud plot of the most commonly occurring keywords. Over the 100 randomly selected gene permutations, protein biosynthesis, 4Fe-4S nucleotidyltransferase and biological rhythms were never found in the 100 random gene permutations gene set.

Individual regions that were enriched for *Bos indicus* content include chromosome 21 (see Data Availability), at positions 7.5–10 Mb with a 6-fold difference in *bosind_250* values between *Bos taurus* and *Bos indicus* (*bosind_250* value 0.67 with *Bos taurus* vs 0.11 with *Bos indicus*). One gene of interest, melanoma antigen family L2 (*MAGEL2*), is implicated in fertility and evidence indicates the gene may influence testicular size, fertility and growth^22^. *MAGEL2* is also involved in circadian rhythms and adaptation to new environments (in mouse knockout studies)^23^.

The region between 53–55 Mb on chromosome 8 is of strong *Bos indicus* origin and contains the genes *PRUNE2*, *GNA14*, *GNAQ*, *CEP78* and *PSAT1*. In Simmental cattle, an association analysis with foreshank weight found a region that includes the genes *GNAQ* and *CEP78*^24^.

On chromosome 14 at positions 24,250,000-24,500,000 bp, the XK, Kell blood group complex subunit-related family, member 4 (*XKR4*) gene is found approximately 396 kb upstream of the *PLAG1* gene. The gene is of interests as it has been observed to be associated with subcutaneous rump fat thickness^25^. In addition, polymorphisms in this region have been found to be associated with rump fat thickness, residual feed intake, average daily feed intake and gain in cattle^25^.

### Regions of the genome that are of *Bos taurus* origin

While *Bos indicus* cattle are well adapted to harsh environments, they can have reduced production performance compared with *Bos taurus* cattle, including slower growth, and reduced fertility (particularly later puberty and stronger expression of *post-partum anoestrus*)^26^. We speculate that the Brahman genome could be enriched for *Bos taurus* segments in regions affecting these traits, reflecting some artificial selection during the grading up process^27,28^. Formation of the Brahman breed involved two different “grading-up” stages, the first was in USA and the second was in Australia. This predominately involved crossbreeding female *Bos taurus* animals with Brahman bulls. This is reflected by the introgression of large segments of *Bos taurus* in the Brahman genome on chromosome X (see Data availability, chromosome X heatmap).

As was done when looking at genes found in regions of significant *Bos indicus* origin, the fixed-size windows were sorted based on the percent difference of the *bosind_250* values in ascending order between *Bos taurus* and Brahman. The top 5% fixed were selected (253 fixed-size windows) and all genes were extracted from these windows. Supplementary Table S2 provides the full list of the top 5% most significant fixed-size windows including all genes and gene information found within each fixed-size window. This resulted in a total of 892 genes that are found in regions with significant *Bos taurus* introgression and used for functional annotation with the DAVID package^16,17^. The results show enrichment for genes involved with olfactory, major histocompatibility complex (*MHC*) I and II, nucleosome core and the G-coupled protein receptor (*GPR*) family (Fig. 3b). As a control, the same random gene selection permutation test was performed as mentioned previously, in which 892 genes randomly selected over a total of 100 permutations and each permutation was functionally annotated with DAVID. Over the 100 random permutations, we found no enrichment for genes associated with *GPR*, olfactory, immune system and chromatin binding (see Data availability). The *GPR* family is of interest because some genes from *Bos taurus* introgressed regions that are enriched for this family of proteins, are associated with puberty. Thyrotropin receptor (*TSHR*) is one example, as SNP found in this gene are associated with fertility traits in cattle^5^ and *TSHR* was identified as a putative domestication gene in chickens^29^. Other genes of interest that are found to be enriched in the *GPR* family include, but are not limited to, *ADGRL3*, *CELSR2*, *QRFPR*, *RRH*, *VIPR1* and the *TAAR* family.

A Gene Ontology (GO) analysis looking at the biological process (BP) found that of the genes in *Bos taurus* introgressed windows, there is a trend for associations with processes involved in immunity, nucleosome assembly, *GPR* family and sensory perception of smell.

### Chromosome 14, *PLAG1* gene and surrounding region

**Figure 4:**
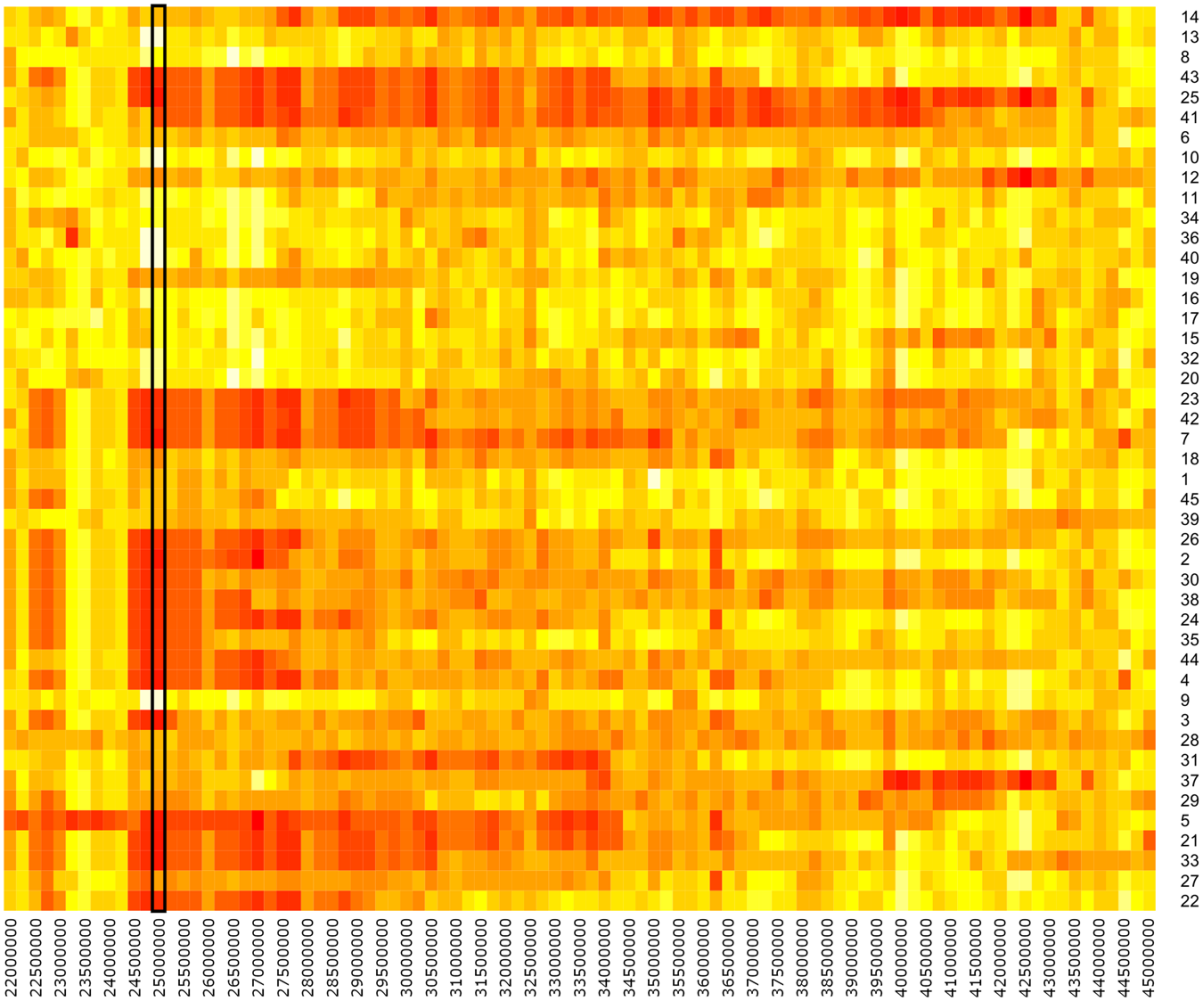
Heatmap of the Chromosome 14 region with high Bos taurus introgression. This includes the PLAG1 gene found at positions 25,007,291–25,009,296 as indicated by the black box. On the y-axis are all the sequenced animals, ordered by year of birth (with older animals at the top). On the x-axis is the genomic positions, incremented in 250 kb windows. A red colour indicates more Bos taurus introgression, a white colour indicates more regions that are of Bos indicus origin.

The most significant *Bos taurus* introgression observed was an extensive genomic region on chromosome 14 around 2.2–4.2 Mb (Fig. 4). This region contains several widely known and well described genes, including the zinc finger protein Pleomorphic adenoma gene 1 (*PLAG1*) gene. This region is of interest as previous GWAS studies have found significant associations between the *PLAG1* gene, including surrounding genes, to have important associations with stature^30^, fertility^26^ and beef production traits, such as carcass weight^26,31–33^. In the study by Fortes *et al*.^26^, there are alleles in this region that have been heavily selected for in Brahmans due to their associations with stature, growth and carcass traits, despite having a negative effect on fertility (later puberty)^26^.

A simulation analysis was performed (Methods) to determine if this large *Bos taurus* introgressed region around the *PLAG1* gene occurred randomly or was the result of selection. The simulation study was performed using the tool SFS_CODE^34^ where two ancestral populations of *Bos taurus* and *Bos indicus* animals were simulated. We then simulated a cross-breeding event between the two ancestral breeds to form a simulated Brahman breed. We calculated the FST using the Weir & Cockerham calculation from vcftools for the simulated and real datasets (Methods). FST between the simulated Brahman and *Bos taurus* populations closely matched the FST between the real Brahman and *Bos taurus* populations (0.25 and 0.26 respectively). In Methods, the simulation analysis is described in more detail. Supplementary Fig. S2 shows a plot comparing the frequency of the *Bos taurus* introgressed segments between simulated and real Brahman animals. In the simulated Brahman data, we find a higher frequency of single fixed-size windows (<250,000 bp) that are of *Bos taurus* origin with a lower frequency of fixed-size windows of *Bos taurus* origin that are larger. Meanwhile, in the real Brahman data, we observe a higher frequency of *Bos taurus* introgressed segments that extend two or more fixed-size windows (>250,000 bp) (see Supplementary Fig. S2). In the case of the large *Bos taurus* introgressed segment surrounding the *PLAG1* gene, there were no segments of *Bos taurus* introgression as long in the simulated Brahman dataset. This provides some evidence that the large *Bos taurus* introgressed segment on chromosome 14 at the *PLAG1* gene is likely the result of artificial selection and not due to random drift.

Other genes of interest in this region include *LYN*^35,36^, *MOS*^35,36^, *CHCHD7*^37^, *PENK*^35^, and *SDR16C5*^35^, which have been proposed in various studies as associated with milk production, reproduction, muscle formation, carcass traits and body size.

We observed that 26 “older animals” (those born between 1953 to 1989) are less likely to have the *Bos taurus* introgression in this region, while 20 of the “younger” animals (those born between 1990 and 2005) carry the *Bos taurus* introgression in this region (Fig. 4).

The *XKR4* gene as discussed previously is found in this region, just 396 kb upstream of the *PLAG1* gene (Fig. 4) and associated with rump fat thickness, residual feed intake, average daily feed intake and gain in cattle^25^.

### Additional genomic regions with *Bos taurus* introgression

The second half of chromosome 8 shows several regions with an increased frequency of *Bos taurus* ancestry (see Data availability). The positions around 75,000,000–81,500,000 bp display some of the most substantial *Bos taurus* introgression on the chromosome. The study by Bolormaa *et al*.^2^ found that the region around 81.4 Mb on chromosome 8 has a high level of *Bos taurus* ancestry compared to other regions on the chromosome. Isolating the genes found in these positions, a DAVID^16^ analysis reveals that the genes are enriched for associations with carbohydrate transmembrane transport, renal water absorption and urea transmembrane transport. One of these genes, β1,4-galactosyltransferase I (*B4GALT1*), encodes an enzyme that adds glycoprotein glycans to oligosaccharides^38^, and in bovine was found to be associated with milk synthesis and secretion in mammary glands. Further, downregulation of the gene is involved in reduced milking frequency and volume^39^. Possibly better milking ability associated with these alleles means higher weaning rates and therefore increased calf survival.

The genes calpastatin (*CAST*) and heat shock protein beta 1 (*HSPB1*) are both located in regions that exhibit substantial *Bos taurus* introgression on chromosome 7 and 25, respectively; *CAST* is a key gene regulating the rate of protein turnover^40,41^ and plays a role in meat quality^42^.

Chromosome 12 and 23 (around the *BoLA* region) show relatively substantial *Bos taurus* introgression at positions 69,000,000-79,000,000 and 22,750,000–34,000,000, respectively. While immune related genes are of great interest in adaptation, this apparent high level of *Bos taurus* introgression could possibly be a cause of assembly errors in the reference genome or due to a high number of copy number variants observed in this region (CNV)^43^.

### Fixed non-reference alleles in the Brahman genome

If a variant in Brahman cattle and *Bos taurus* is fixed for the alternative allele, it may indicate that this variant has been under strong selection because they confer an advantage for adaptation to harsh environments (although this can happen through drift as well, and this analysis does use Brahman as a proxy for *Bos indicus*). These mutations can occur for at least two reasons. Firstly, a mutation arose in the ancestral animals (before the split between *Bos taurus* and *Bos indicus*) and one allele was fixed in one or more of the four *indicus* foundation breeds while the other allele was fixed in *Bos taurus* due to either genetic drift or natural or (less likely) artificial selection. Secondly, mutations may have occurred spontaneously in the Brahman population following which heavy selection has occurred. There were 20,917 SNPs fixed for alternative alleles in the Brahman and *Bos taurus* (1000 bull genomes^10^) genomes.

Annotation of fixed variants show relatively similar proportions to the annotations of all genomic Brahman variants (67.32% intergenic and 25.35% intronic). However, we find an increased proportion of missense mutations in SNP that are fixed for the alternative allele in Brahmans versus *Bos taurus*. 0.86% of Brahman SNP that are fixed for the alternative allele are annotated as missense, this value varies from the proportion of Brahman genomic SNPs annotated as missense, which is 0.35% (see Table 1 and Supplementary Table S1). This difference in the proportion of missense annotated SNP represents an 85% increase in the percent difference between fixed variants for the alternative allele that are missense, compared to all missense variants in the WGS dataset. A chi squares analysis reveals that this difference is significant, with a chi-square score of 29.629 and a *p-*value of 0.009 making the difference significant at *p* < 0.05.

**Table 1:** Annotation information of the variants that are fixed in Brahmans for the alternative allele.

Annotation	No. of SNP	Percent of total	Percent difference*
3_prime_UTR_variant	56	0.268	34.074
5_prime_UTR_variant	23	0.11	87.260
coding_sequence_variant	10	0.048	196.387
downstream_gene_variant	1,200	5.737	68.453
intergenic_variant	12,066	57.685	−15.416
intron_variant	5,775	27.609	8.543
missense_variant	180	0.861	85.524
non_coding_transcript_variant	9	0.043	39.074
splice_acceptor_variant	3	0.014	157.820
splice_donor_variant	3	0.014	157.125
splice_region_variant	44	0.21	104.743
stop_gained	3	0.014	94.681
synonymous_variant	83	0.397	−41.754
upstream_gene_variant	1,429	6.832	71.557
Unknown	33	0.158	195.742
Total	20,917	100	

The percent change with all variants, represents the different (represented as a percentage) between the number of variants found in each functional class that are fixed, and the total number of variants found in each class.

*Percentage difference indicates the difference in the percent between the fixed variants in a class with the total number of variants found in that class (see Supplementary Table S1).

Some genes with missense mutations fixed in Brahmans for the alternative allele stand out, including *IRGQ*, *KIR3DS1*, *ATG9B*, *ADGRB2*, *PRAME*, *CMPK2*, *CLEC4G* which are all related to immunity. *ATG9B* is one of the main genes involved in autophagy and has been found to be associated with initiating autophagy^44^. *CMPK2* is believed to be associated with macrophage terminal differentiation and was recently found to be a candidate gene for association with lower weight gain and increased viraemia levels in response to porcine reproductive and respiratory syndrome virus infection while showing evidence of allele specific expression in pigs^45^. These findings suggest mutations associated with immunity have become fixed in the Brahman breed, and could be one reason why the Brahman breed is so resilient and resistant to a range of pathogens that affect *Bos taurus* breeds.

The full list of SNP can be found in Supplementary Table S3.

### Brahman Polled mutation

Four additional Brahmans that were polled (do not grow horns), identified through progeny testing, were sequenced. One animal (47) was heterozygous for the polled allele (*Pp*) and the other three animals (48, 49, 50) were homozygous for the polled allele (*PP*). The three *PP* animals were compared to the 46 Brahmans (as used in this study), in which we refer to as the 46 original animals. The 46 original animals were horned (*pp*) animals (although there is a remote chance that an animal might be *Pp* and was not recorded as such).

The polled allele has been located on chromosome 1 in the positions 1.6–2.2 Mb in cattle^46,47^ and also in composite cattle^48^. Three putative mutations have been reported. The first polled mutation is an 80 kb duplication in the region on 1.91–1.99 Mb that is found in Friesian cattle^8^. The second polled mutation is found starting at the position 1.71 Mb and is believed to be of Celtic origin, described as a 212 bp duplication in polled animals that replaces a 10 bp sequence at the positions 1,706,051–1,706,060 bp^7^. This mutation is referred to as the Celtic mutation. The third polled mutation originates from Mongolian Turano cattle and was recently found to be introgressed in Mongolian Yaks^3^. As with the other two polled mutations, this mutation is found on chromosome 1 between positions 1,889,854 bp and 2,010,574 bp. The architecture of this mutation is an 11-bp motif (conserved among Bovidae and duplicated in the Friesian mutation) due to a rearrangement of a 219 bp duplication-insertion, a 7 bp deletion and a 6 bp insertion^3^. This third polled mutation was not observed to be segregating in the Brahman animals, and it is unlikely to be segregating as it has only ever been described in Mongolian Turano cattle and Mongolian yaks.

In Brahmans, a single study has mapped the polled locus in 68 genotyped Brahman cattle to the same region as in *Bos taurus* cattle^49^, yet the origin of the Brahman polled mutation remains unknown.

Examining the fixed-size windows in the polled locus region, shows slight introgression of *Bos* taurus (see data availability, Chromosome 1). Using our sequence data, the total number of reads aligning to the UMD 3.1 reference were counted in 50 bp incrementing windows in and around the polled region and the standard deviation was calculated for each of the 50 bp window (see Methods). We examined the coverage of the reads because if an increase in the coverage occurs, it can indicate a duplication mutation. In this study, the region around 1.65 Mb and 1.90 Mb stands out as an increase in coverage for the three *PP* animals compared to the 46 original Brahman bulls, indicating the presence of structural variants, such as duplications, in the *PP* animals.

**Figure 5:**
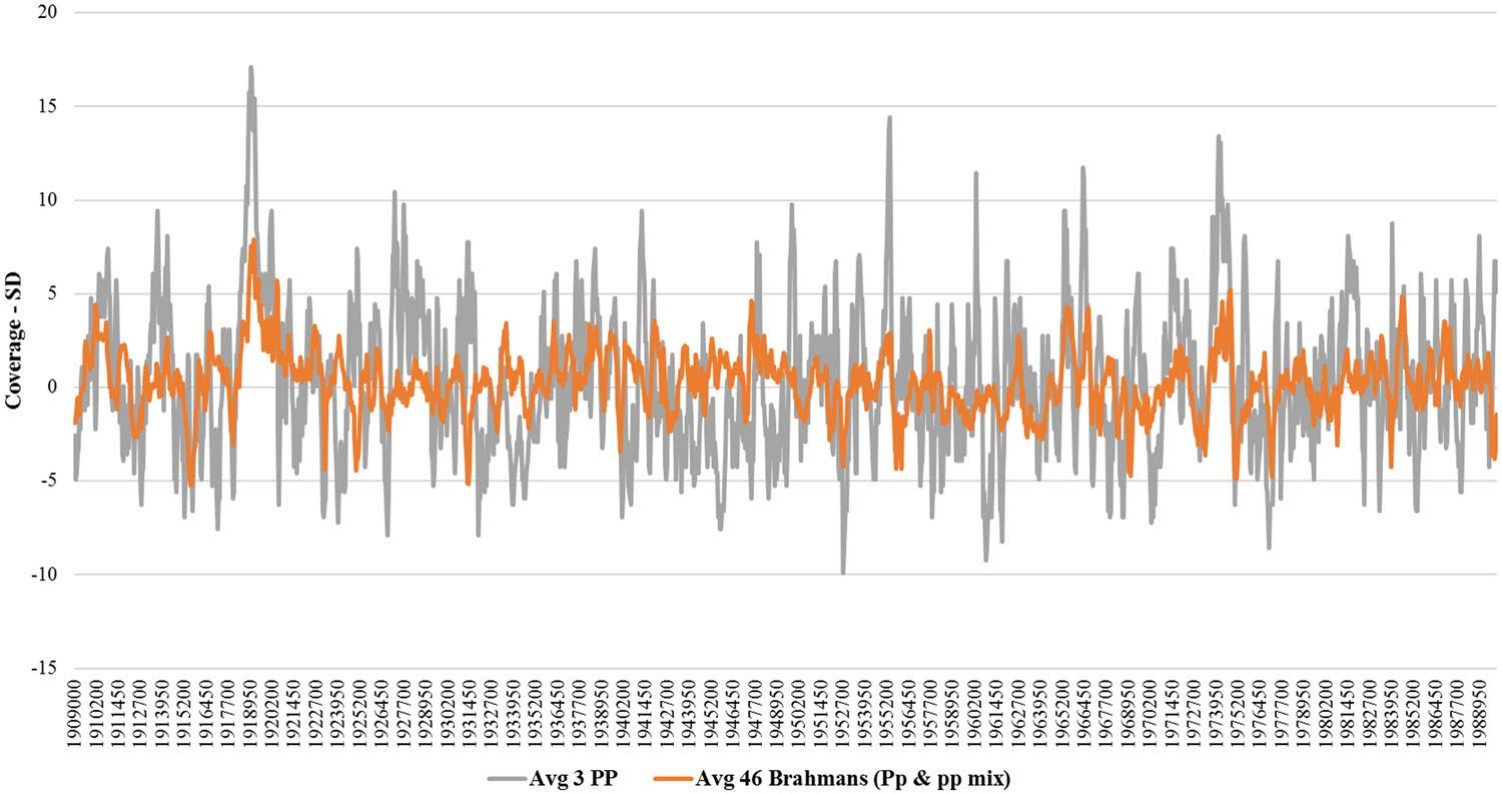
Holstein-Friesian 80 Kb polled variation. Figure showing the read coverage (expressed as standard deviations from the mean) between the three homozygous polled Brahmans and the 46 original Brahman animals in the region where the 80Kb Holstein-Friesian polled duplication is found.

In the positions where the Friesian mutation is on chromosome 1, we find no distinct difference or patterns in read coverage across the entire 80 Kb region between the three *PP* animals and the 46 original animals (Fig. 5). If the Friesian 80 Kb duplication was present in Brahman, we would expect to find at least a two-fold increase in the coverage where the mutation exists between the homozygous polled and the 46 original Brahmans. Thus, in this study, we did not find evidence that the Friesian mutation is segregating in the three *PP* polled Brahman animals.

**Figure 6:**
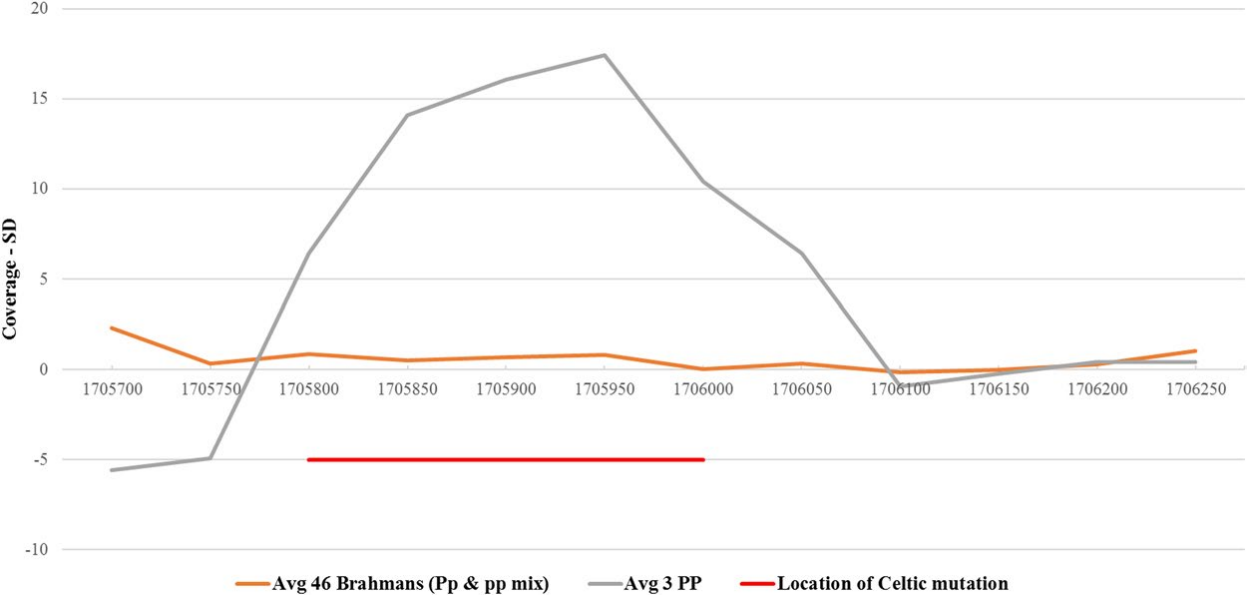
Plot of the number of reads mapping to the reference homed in on where the Celtic mutation has been described. In the 3 PP animals, we see an increase in the coverage, characteristic of a duplication event, while the 46 original animals show consistent coverage. Here the orange line represents the standard deviation in the coverage for the original 46 animals. The grey line represents the standard deviation in the coverage for the 3 PP animals. The red line represents the location of the Celtic mutation as described by Wiedemar *et al*.^7^.

At the positions on chromosome 1 where the Celtic mutation is predicted to be^7^, we find that the 3 *PP* animals show an increase in coverage, when compared to the 46 original Brahman (Fig. 6). In Fig. 6, we see that a duplication is possibly found at this region, around where the actual Celtic mutation is described, as the coverage for the *PP* animals is increased significantly.

Furthermore, the BAM files containing the mapped reads for chromosome 1 were visualized using the Integrative Genomics Viewer (IGV) tool concentrating on the region where the Celtic mutation is found. We find in the three *PP* animals, there is a region between the positions of 1,706,045–1,706,060 bp which shows a 10 bp deletion event, consistent with the Celtic mutation. This deletion can be unambiguously seen in two of the polled animals (48 and 50) (see Supplementary Fig. S3), with minimal reads mapping to this region, especially for these two polled animals. Furthermore, the *PP* animal 49 seems to be carrying some variant of the polled mutation, as few reads map to the 10 bp deletion. This can possibly indicate that while this animal is homozygous for the polled mutation, the mutation affecting polledness might not be at the 10 bp region where the deletion is located. This needs to be followed up with more research and will provide the basis for future work.

In the 46 original animals that had their genomes sequenced (Data Repository), most do not display this deletion, indicating *pp (*horned) animals with a small chance of *Pp* animals.

## Conclusion

There has been clear introgression of *Bos taurus* alleles into Brahman in several regions, with the largest region on Chromosome 14 in the *PLAG1* region. This introgression could have been driven by selection for increased stature. The regions of the Brahman genomes with near fixation of indicine alleles were enriched for genes associated with protein synthesis, which could reflect selection for reduced protein turnover to cope with harsh environments. However, in general, we found relatively few regions completely fixed for *Bos indicus* content, which suggests adaptation might be a highly polygenic trait (affected by a large number of loci of small effects). It also should be pointed out that because the Brahman genome is largely *Bos indicus* in origin, we had much lower power to differentiate genomes regions enriched for *Bos indicus* content by chance *versus* enriched by selection. Finally, we provide evidence to suggest that the polled mutation in the Brahman cattle of our study, at least in the individuals we had, is a result of the Celtic mutation rather than the Holstein-Friesian mutation.

## Methods

### Animal ethics statement

DNA was extracted from commercially collected semen straws or Ampoules that have previously been collected for commercial purposes and these were purchased or donated to the project.

### Sequence data

Sequenced bulls were selected using an algorithm that identified 46 bulls that captured the highest proportion of genetic variation in the breed. The algorithm is based on an analysis of an extensive Brahman pedigree and a stepwise regression procedure to avoid double counting of ancestral genomes. Furthermore, the algorithm considered whether DNA, extracted from commercially collected semen straws or Ampoules, was available for a bull or not^9,10^. Three additional bulls that were known to be homozygous for the polled phenotype were also analysed in this study.

The selected bulls were sequenced on an Illumina HiSeq sequencer, at an average of 12.5 times genome coverage and a range of 10–30 times genome coverage. Data was trimmed and filtered using the tool Trimmomatic^50^, merging singleton reads (reads where one of the matching paired ends passed quality filtering) into a single file. We filtered for reads that had a minimum length of 36 and an average quality of 20.

### Variant calling

After quality control and trimming, reads (including the singleton reads) were mapped to the bovine genome (UMD3.1) with BWA MEM^51^. All SAM files were converted to BAM format, sorted based on their coordinates and duplicates were marked using the Picard tools Sortsam, and MarkDuplicates^52^. The GATK^12^ tool IndelRealigner was used to perform a local realignment of the reads around Indels and the Picard tools MarkDuplicates was additionally used to remove all duplicates in the final BAM files. BAM index files were generated using the Picard tool BuildBamIndex.

Variant discovery was performed using GATK following the GATK Best Practises manual^12^. The GATK^12^ tool HaplotypeCaller was used to call all raw variants using the *Bos taurus* reference genome UMD3.1 and all raw variant VCF files were joined via the GenotypeGVCF tool to produce a single VCF file. The variants include single nucleotide polymorphisms (SNP) and small insertion/deletions (Indels). SNP and Indels were filtered using hard filters with thresholds that were based on what is recommended by GATK^53^ for removal of variants with poor quality scores. VQSR was not used in this study for filtering of variants.

SNP were filtered with the following annotations and thresholds:

$${\rm{QD}} < 2.0\parallel {\rm{FS}} > 60.0\parallel {\rm{MQ}} < 40.0\parallel {\rm{ReadPosRankSum}} < -\,8.0\parallel {\rm{MQRankSum}} < -\,{\rm{12.5}}\parallel {\rm{SOR}} > 3.0$$

Descriptions of each filter can be found in the GATK Best Practises manual^12^. In brief, QD is the quality by depth which is the quality score normalized by allele depth. FS is the Fisher Strand test to detect strand bias, lower scores indicate more strand bias. MQ is the root mean square of the mapping quality score. ReadPosRankSum is the ranked sum test for the distance of alleles from the end of the reads, as the closer the variant is to the end of the read, the more error prone it is. MQRankSum looks at the mapping qualities of reads that support the reference allele with those that support the alternative allele and SOR measures strand bias. InbreedingCoeff measures the level of inbreeding in a group of samples by estimating the population allele frequency from the sample genotypes.

The threshold to hard filter Indels as used in this study is:

$${\rm{QD}} < 4.0||{\rm{FS}} > 100.0\parallel {\rm{InbreedingCoeff}} < -\,0.8||{\rm{ReadPosRankSum}} < -\,10.0||{\rm{SOR}} > 6.0$$

### *bosind_250* calculation

BTA and Gir variants were obtained from the 1000 bulls genomes project^10^ and from the study by Liao *et al*.^15^, respectively. Only SNP data from each study was used and allele frequencies were calculated using in-house scripts. Following this, SNP found in common between *Bos taurus* and Brahman and between Gir and Brahman were determined, selected and placed into separate files. This was done with a python script that selected SNP that shared the same genomic location.

To determine introgression of *Bos taurus* and Gir genomic DNA in Brahman, a similar method was inspired by the calculation as described in Bolormaa *et al*.^2^, with the difference being our formula uses 250 kb windows. Our method involved calculating *bosind_250* values, where the *bosind_250* statistic estimates how differentiated two populations are based on sequence, allele frequency and SNP calls. In this analysis *bosind_250* values were calculated between *Bos taurus* and *Bos indicus* SNP that are found in the Brahman animals using the following formula:

1 $${b}{o}{s}{i}{n}{d}{\rm{\_}}250=\frac{Ht-Hs}{Ht}$$

where:

2 $$Hs=PBT(1-PBT)+Pbrai(1-Pbrai)$$

and

3 $$Ht=\frac{2\ast (PBT+Pbrai)}{2}\times 1-\frac{PBT+Pbrai}{2}$$

*PBT* is the SNP allele frequency of the alternative allele in either *Bos taurus* or *Bos indicus* and *Pbrai* is the SNP call in that Brahman SNP individual. *Pbrai* is 0 if the SNP call is homozygous for the reference, 0.5 if the SNP call is heterozygous and 1 if the SNP call is homozygous for the alternative allele.

The calculation was performed twice for each Brahman animal across all SNP, the first calculation used common SNP between Brahman and *Bos indicus*, the second calculation used common SNP between Brahman and *Bos taurus*.

All SNPs were grouped into windows of 250 kilobases (kb) and the average *bosind_250* values (*bosind_250avg*) were calculated for each window We used 250 kb windows as this provides enough SNP from sequence data in each window to perform this analysis and obtained robust estimates of the *bosind_250* value. This was done by adding the *bosind_250* values across all SNP within a 250 kb window and dividing that number by the total number of SNP found in that window as shown in the following formula:

4 $$bosind{\rm{\_}}250avg=\frac{{\sum }^{}({b}{o}{s}{i}{n}{d}{\rm{\_}}250fw)}{n}$$

where *bosind_250fw* are the *bosind_250* values for all SNP found in a fixed-size window and *n* is the total number of SNP found in that fixed-size window.

### SNP Annotation

Annotation of the Brahman SNP was carried out using the NGS-SNP tool^14^. CpG Isles annotations were from the study by Su *et al*.^54^. Micro RNA (miRNA) target site annotations were from the Microcosm Database^55^. Long noncoding RNA annotations were from the paper by Koufariotis *et al*.^56^.

### Simulation study

A simulation study was performed to investigate if large *Bos taurus* introgressed segments in the Brahman genome (such as *Bos taurus* introgression found around the *PLAG1* gene on chromosome 14) could be due to chance, given the history of the breed, or are due to selection (e.g. very unlikely to have occurred by chance).

To perform this analysis, we first simulated an ancestral founder population using the simulation software tool SFS_CODE^34^. After 100,000 generations we split the ancestral population into two separate populations. The first population was a simulated *Bos taurus* group and the second population was a simulated *Bos indicus* group. After 10,000 generations, from the simulated founder *Bos taurus* and *Bos indicus* populations, we formed a Brahman population by simulating a cross-breeding event between the *Bos taurus* and the *Bos indicus* populations. The cross-breeding event simulated the “grading up process” of Brahman cattle. After 30 generations, the final Brahman population was simulated with an FST that closely matches the FST in the real Brahman animals. No selection was assumed in the formation of the simulated Brahman breed and the simulation of the founder *Bos taurus* and *Bos indicus* breeds.

For the tool SFS CODE, the following parameters were used: A low mutation and recombination rate of 0.01 per each locus of 10,000 kb. The population size for the ancestral *Bos taurus* and *Bos indicus* breeds was set at 10,000. The Brahman population was simulated to occur after 30 generations of crossbreeding the simulated *Bos taurus* and *Bos indicus* populations. This resulted in a total of 1,000 Brahman animals.

We calculated the FST between the simulated Brahman and the simulated *Bos taurus* animals using VCFtools^57^ with the following command:

$$\begin{array}{c}{\rm{vcftools}}\mbox{--}{\rm{vcf}}\,{\rm{simulated}}\_{\rm{brahman}}.{\rm{vcf}}\mbox{--}\mathrm{weir} \mbox{-} \mathrm{fst}\\  \mbox{-} \mathrm{pop}\,{\rm{simulatedBrahanPopulation}}{\rm{.txt}}\mbox{--}\mathrm{weir} \mbox{-} \mathrm{fst}\\  \mbox{-} \mathrm{pop}\,{\rm{simulatedBos}}\,{\rm{TaursPopulation}}{\rm{.txt}}\mbox{--}{\rm{out}}\,{\rm{brahman}}\_{\rm{vs}}\_{\rm{bosTau}}\_{\rm{fst}}\end{array}$$

The FST between the simulated Brahman and *Bos taurus* animals was 0.25 which closely matches the 0.26 FST between the real Brahman and *Bos taurus* animals. Both FST values were obtained using the Weir and Cockerham calculation from vcftools. The level of differentiation was achieved by tuning the generations of separation between *Bos taurus* and *Bos indicus*.

The genotypes from the vcf files for animals in both the Brahman and *Bos taurus* simulated populations were used to determine the *Bos taurus* introgression in the simulated Brahman breed, as was described earlier in Methods.

Finally, to examine the chance of a long *Bos taurus* introgressed segment (that spans more than one fixed-size window of *Bos taurus* origin), we calculated the length of *Bos taurus* introgressed chromosomal segments in the Brahman genome. This was done by calculating consecutive fixed-size windows with *Bos taurus* introgression across the Brahman genome, both for the real and simulated Brahman data. Next, the frequency of each *Bos taurus* introgressed segments was determined in both the simulated and real Brahman animals. Supplementary Fig. S2 shows a plot comparing the frequency of the *Bos taurus* introgressed segments between the simulated and real Brahman datasets.

### Fixed alleles

Using in-house scripts, we identified the SNPs in which every Brahman animal was homozygous for the alternative allele. In the case of the VCF files, when all animals had (1/1). The SNP were compared to *Bos taurus* SNP from the 1000 bulls genome project^10^. Only the SNP that were unique to Brahman and not found in the *Bos taurus* dataset were kept. These SNP are said to be fixed in the Brahman and not in *Bos taurus*. As annotation was carried out with the NGS-SNP tool^14^, we identified the SNP that had a missense mutation annotation.

## Electronic supplementary material

Supplementary Figures

Supplementary_Table_S1

Supplementary Table S2

Supplementary_Table_S3

## Data Availability

The datasets generation during analysis for the current study are available at NCBI BioProject database with project number: PRJNA432125. This includes all the BAM files for the 46 sequenced bulls and the three additional bulls that are known homozygous polled. Heatmaps for all 29 autosomal chromosomes that show the *Bos taurus* and *Bos indicus* introgression are hosted by LabArchives, LLC (http://www.labarchives.com/) with 10.6070/H4RJ4H2D. All files are in pdf format
