# Supplementary material for: Sequencing the mosaic genome of Brahman cattle identifies historic and recent introgression including polled
Source: Sci Rep. 2018 Dec 10;8:17761. doi: 10.1038/s41598-018-35698-5 (PMC6288114; doi:10.1038/s41598-018-35698-5)
Supplement: Supplementary file 1 — Supplementary Figures [file 41598_2018_35698_MOESM1_ESM.pdf]

Supplementary Figures

Sequencing the mosaic genome of Brahman cattle identifies historic and recent introgression including polled

L. Koufariotis[1*], B.J. Hayes[1], M. Kelly[2], B. M. Burns[3], R. Lyons[4], P. Stothard[5], A.J. Chamberlain[6] and S. Moore[1]

[1]Centre of Animal Science, Queensland Alliance for Agriculture and Food Innovation, The University of Queensland, Brisbane, Queensland, 4072, Australia
[2]Australian Agricultural Company (AACo), Brisbane, Queensland, 4006, Australia
[3]Department of Agriculture and Fisheries, Rockhampton, Queensland 4702, Australia
[4]School of Veterinary Science, The University of Queensland, Gatton, Queensland, 4343, Australia
[5]Department of Agricultural, Food and Nutritional Science, University of Alberta, Edmonton, Alberta T6G 2C8, Canada
[6]Agriculture Victoria, Agribio, Centre for Agribiosciences, 5 Ring Road, Bundoora, Victoria 3086, Australia

Email addresses of authors:

LK: r.koufariotis@uq.edu.au
BJH: b.hayes@uq.edu.au
MK: mkelly@aaco.com.au
BMB: Brian.Burns@daf.qld.gov.au
RL: r.lyons2@uq.edu.au
PS: stothard@ualberta.ca
AJC: amanda.chamberlain@ecodev.vic.gov.au
SM: s.moore3@uq.edu.au

*Corresponding Author: Lambros Koufariotis. Email: r.koufariotis@uq.edu.au
Phone: +61 7 334 62179
Address: Building 80, 306 Carmody Road, The University of Queensland, St Lucia, QLD, 4072, Australia

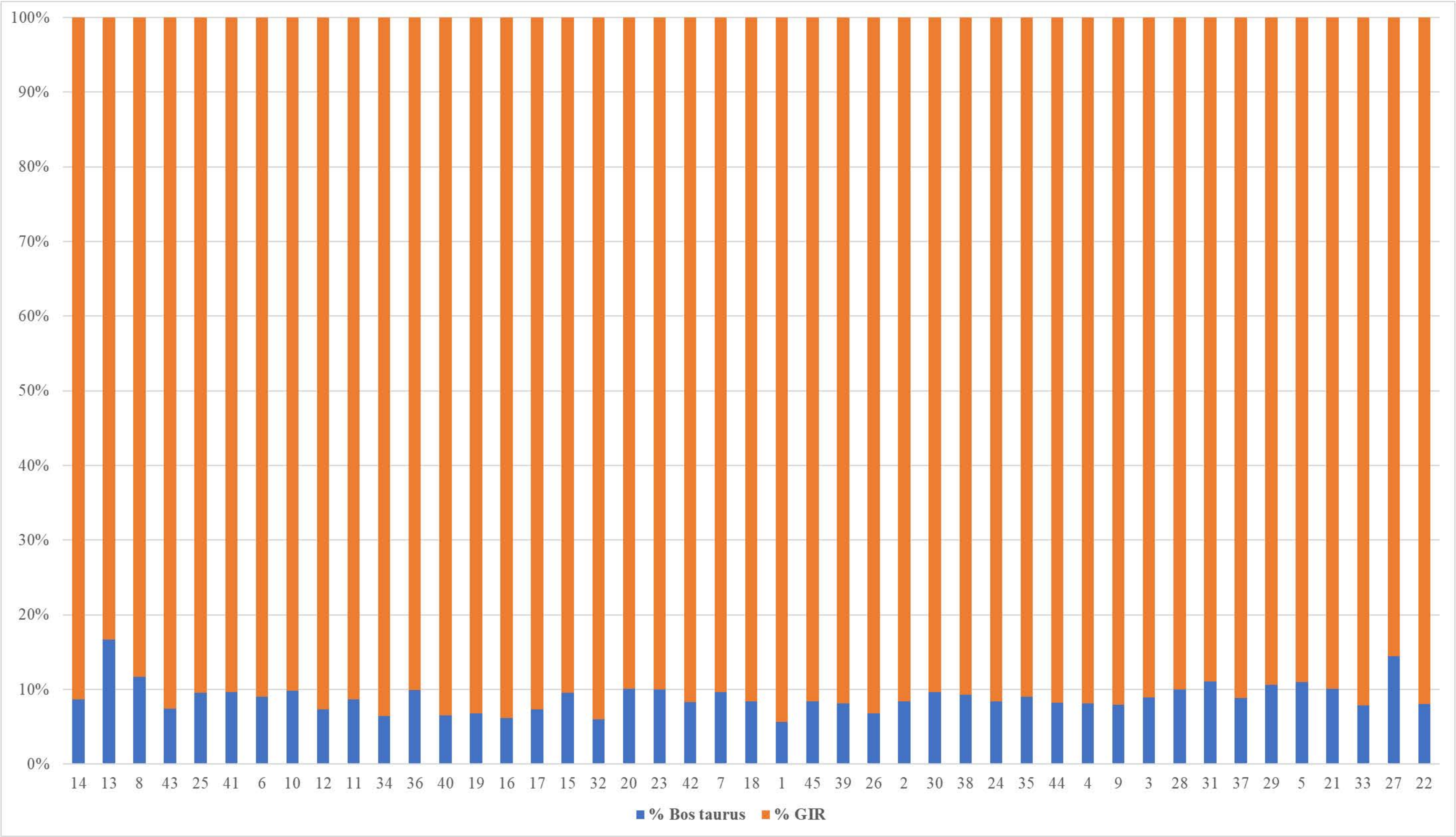

Supplementary Figure S1. Plot of the percent of *Bos taurus* content for each sequenced animal. Blue represents the percent of *Bos taurus* and orange represents indicine or unclear (FST for *Bos taurus*/Brahman and Gir/Brahman is too similar) regions. *Bos taurus* content was determine by calculating the fixed windows that had the top 5% most significantly large percent difference in the FST between *Bos taurus*/Brahman and Gir/Brahman with the FST for *bos Taurus* being closer to 0. The y-axis is the total percent of either *Bos taurus* or indicine/unclear regions and the x-axis represents the sequenced animals. Animals are sorted based on date of birth, from oldest to youngest.

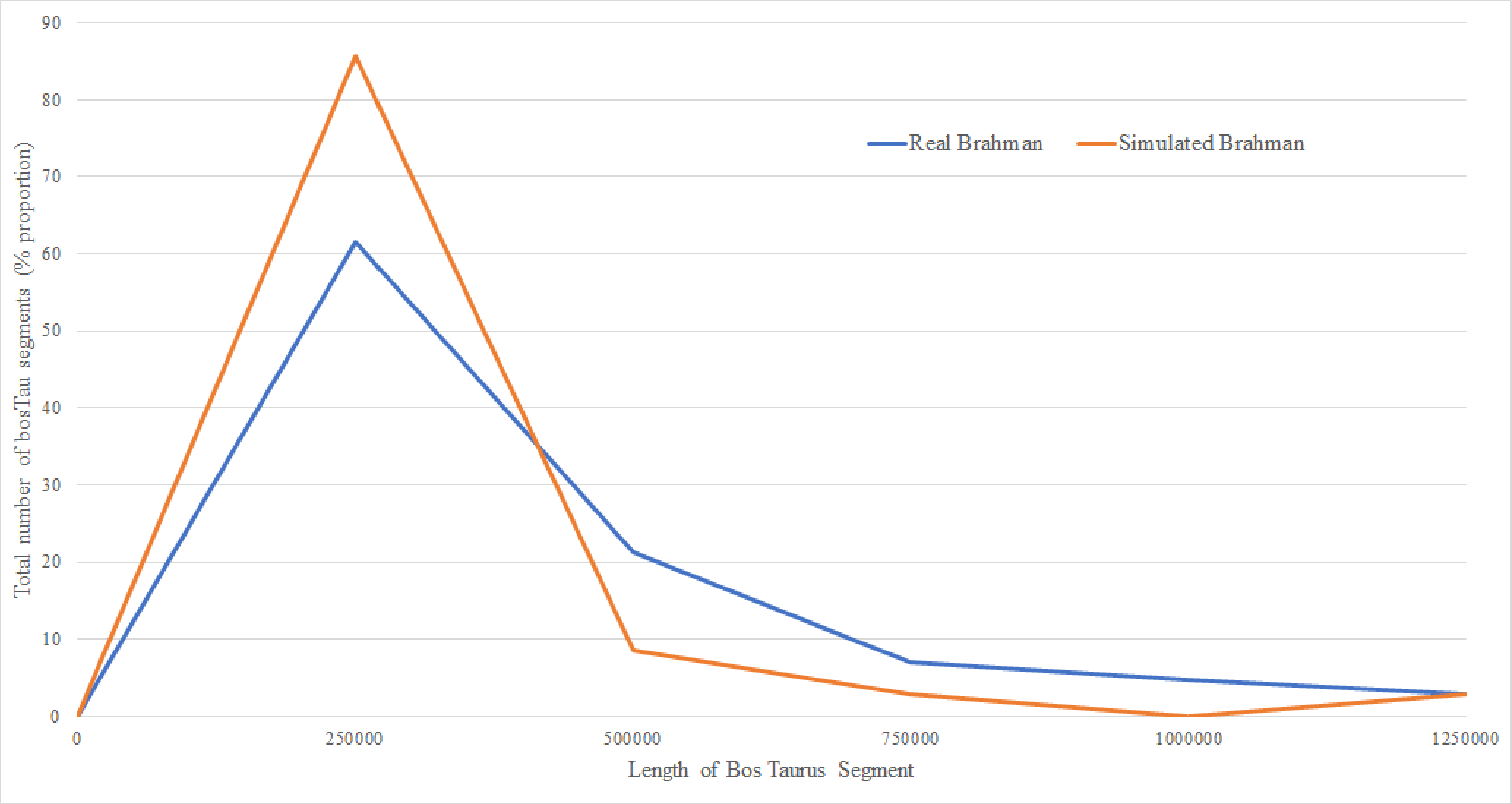

Supplementary Figure S2. Plot of the length of *Bos taurus* chromosomal segments (each 250,000 represents a single fixed window) on the x-axis with the frequency the *Bos taurus* segment is found in the genome (represented as a percent in proportion to total *Bos taurus* segments). The orange plot represents the simulated Brahman animals and the blue plot represents the real Brahman animals. Here, we can see that in the simulated animals there is a higher frequency of shorter *Bos taurus* introgressed segments (<250,000 bp) whereas in the real Brahman data, there is greater frequency of larger *Bos taurus* introgressed segments (>250,000bp).

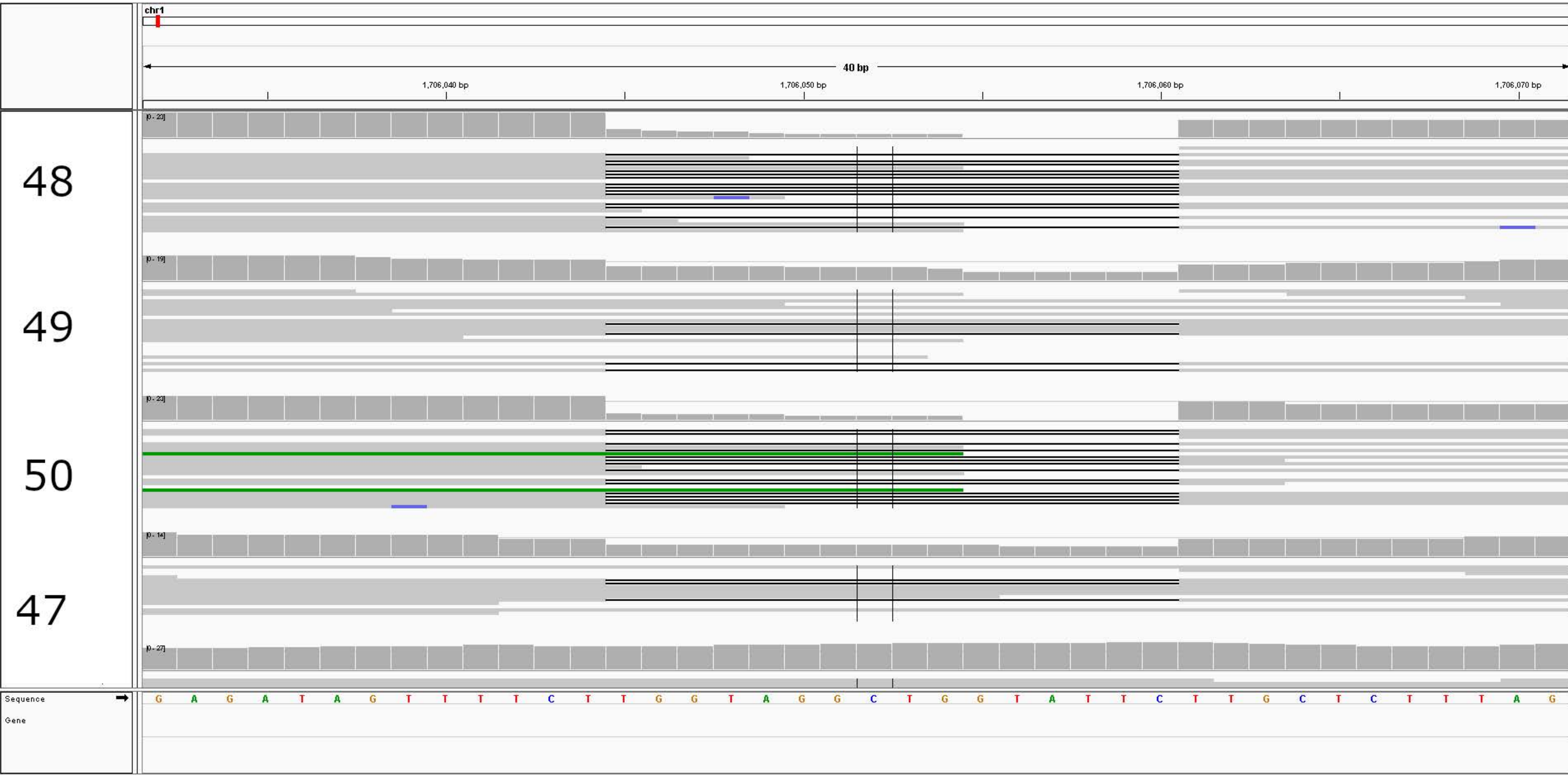

Supplementary Figure S3. Figure showing where the 10 bp deletion is found as seen in the IGV tool when zooming in the positions of where the Celtic mutation is described. The deletion is found between 1,706,045 – 1,706,060 bp on chromosome 1. The thick horizontal black lines, represent unmapped reads. The 3 *PP* animals have the ID 48, 49, 50. The animal 47 is *Pp* polled.
